# Can eye-tracking help to create a new method for X-ray analysis of rheumatoid arthritis patients, including joint segmentation and scoring methods?

**DOI:** 10.1371/journal.pdig.0000616

**Published:** 2024-10-07

**Authors:** Baptiste Quéré, Léonie Méneur, Nathan Foulquier, Hugo Pensec, Valérie Devauchelle-Pensec, Florent Garrigues, Alain Saraux

**Affiliations:** 1 Department of Rheumatology, CHU Brest, France; 2 Université de Bretagne Occidentale (Univ Brest), France; 3 INSERM (U1227), LabEx IGO, France; 4 Medical Information Department, Health Datawarehouse, CHU Brest, France; 5 Department of Radiology, CHU Brest, France; Curtin University, AUSTRALIA

## Abstract

Reading hand and foot X-rays in rheumatoid arthritis patients is difficult and time-consuming. In research, physicians use the modified Sharp van der Heijde Sharp (mvdH) score by reading of hand and foot radiographs. The aim of this study was to create a new method of determining the mvdH via eye tracking and to study its concordance with the mvdH score. We created a new method of quantifying the mvdH score based on reading time of a reader monitored via eye tracking (Tobii Pro Lab software) after training with the aid of a metronome. Radiographs were read twice by the trained eye-tracking reader and once by an experienced reference radiologist. A total of 440 joints were selected; 416 could be interpreted for erosion, and 396 could be interpreted for joint space narrowing (JSN) when read by eye tracking (eye tracking could not measure the time spent when two pathological joints were too close together). The agreement between eye tracking mvdH Sharp score and classical mvdH Sharp score yes (at least one erosion or JSN) versus no (no erosion or no JSN) was excellent for both erosions (kappa 0.97; 95% CI: 0.96–0.99) and JSN (kappa: 0.95; 95% CI: 0.93–0.097). This agreement by class (0 to 10) remained excellent for both erosions (kappa 0.82; 95% CI: 0.79–0.0.85) and JSN (kappa: 0.68; 95% CI: 0.65–0.0.71). To conclude, eye-tracking reading correlates strongly with classical mvdH-Sharp and is useful for assessing severity, segmenting joints and establishing a rapid score for lesions.

## Author summary

Reading of radiography of the hand and foot is the gold standard for evaluating structural joint damage in rheumatoid arthritis anomalies. These radiography are evaluated in a binary manner (erosion and/or joint narrowing per site), or with scores such as the modified van der Heijde-modified Sharp score (mvdH Sharp). The mvdH score is the best way to quantify the evolution but it is a complex, time-consuming and challenging task that requires years of training and experience. The objective of this study was to create a new method of determining the modified mvdH score via eye tracking and to study its concordance with the mvdH score measured by an experienced radiologist for assessing hand and foot involvement in rheumatoid arthritis patients. We found that eye-tracking reading correlates strongly with classical mvdH-Sharp and is useful for assessing severity, segmenting joints and establishing a rapid score for lesions. It would be a more intelligible and playful technique to illustrate the severity of lesions to students, professionals or patients. Our findings may have important implications for improving interdisciplinary communication and collaboration in the diagnosis and treatment of rheumatological disorders. In the future, this technique could also be developed for other disciplines using scores.

## Background

Rheumatoid arthritis is a chronic systemic autoimmune disease mostly characterized by inflammation causing cartilage damage and erosive symmetrical polyarthritis with pain, functional limitations, chronic inflammation of synovial joints and permanent joint damage in the hands. RA affects approximately 0.5–1% of people worldwide [1], and the pace of population ageing, including the rate of rheumatoid arthritis, is growing faster worldwide. A chronic autoimmune disease is often associated with significant morbidity and high costs of care [2,3]. All these factors could have important socioeconomic impacts [4].

To prevent chronic RA, it is important to detect inflammatory signs in the early phase but also to do a follow up to evaluate the evolution [5]. To note, X-rays do not show inflammatory signs, but rather their consequences, which sometimes evolve at a low level. Advances in the field of automatic image processing and analysis have paved the way for automatic detection and diagnosis of arthritis based on how the grade of the synovial region is designed.

Artificial intelligence (AI) can be defined as a computer’s ability to perform a task, usually requiring human intelligence. In the medical field, AI allows self-assessment of patients; warning of drug interactions; interpretation or reconstruction of images; diagnosis and prediction; and literature review [6]. As an algorithmic subgroup of AI, deep learning can generate knowledge with unstructured complex data such as images and improve the efficiency of some clinical tasks. In the brain, deep learning functions like layers of interconnected neurons receive a learning signal (input data) and present output data. The dataset is adjusted with weights and bias. Eye tracking is an artificial intelligence (AI) technology used to measure and study the range of eye movements of participants, including physicians’, in front of an image [7,8]. In the literature, eye-tracking technology has been described to measure cognitive load. Currently, it is used in marketing [9] and in many medical fields [10,11,12]. Eye tracking procedures are valuable tools for investigating the visual diagnostic process in radiology [13] and for evaluating reading interpretations according to the levels of medical expertise [14,15]. Consequently, the required search time also decreases with increasing expertise. Eye tracking allow to analyse fixation durations, the number of fixations on relevant or normal areas, saccades, and image coverage. With increasing experience, it is assumed that saccades expand, influenced by a broader visual range [16,17]. Finally, eye tracking can be used to study cognitive development and plasticity by pupil dilation and spontaneous blink rate [18].

Radiography has been the gold standard for evaluating structural joint damage since it was first proposed by Steinbrocker et al. X-rays of the hand and foot are necessary to detect rheumatoid arthritis anomalies [19]. Hand and foot radiographs in rheumatoid arthritis patients are evaluated in several ways in a binary manner (erosion and/or JSN per site), with scores such as the Sharp score or the modified van der Heijde-modified Sharp score (mvdH Sharp) (73% of trials between 1994 and 2020) ([20,21]. This is a complex, time-consuming and challenging task that requires years of training and experience. Compared with statistical healthy shape models, deep learning can determine deviations that help predict very early progression of RA with possibly higher sensitivity, accuracy, specificity, and precision than human observers [22,23].

The objective of this study was to create a new method of determining the modified Sharp van der Heijde (mvdH) score via eye tracking and to study its concordance with the mvdH score measured by an experienced radiologist for assessing hand and foot involvement in rheumatoid arthritis patients. Eye tracking reading could help to segment the joints and establish quickly a score of lesions with the fixation time score and pixels at the same time.

## Methods

### Readers

**Fig 1:**
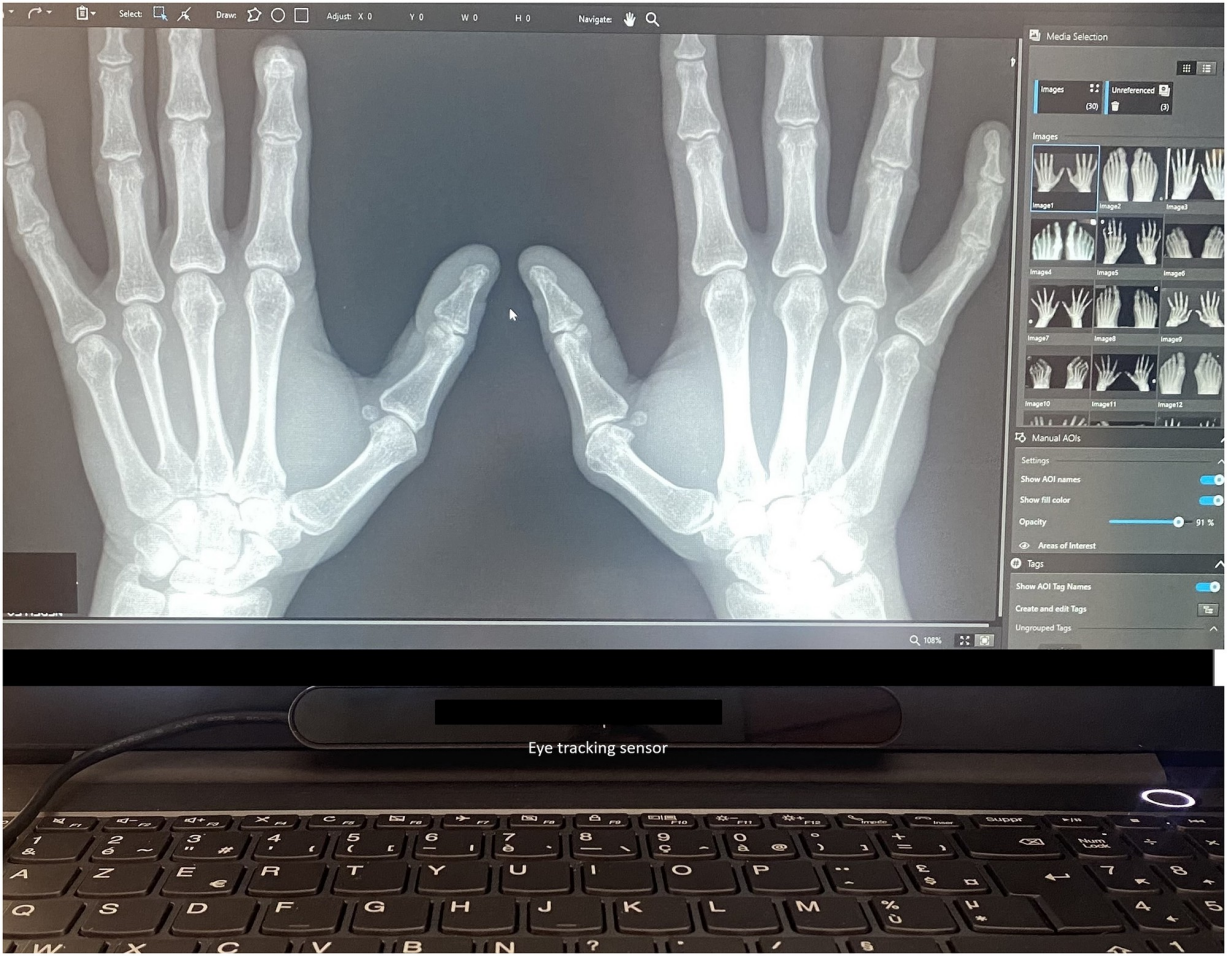
Screen shown by readers using the eye-tracking software. The eye-tracking sensor below the screen capture the eye chips induced by illuminators.

Our study included a medical student (reader 1) trained in the mvdHSharp lecture and the eye-tracking lecture who read radiographs via eye tracking and an experienced radiologist who read only the mvdHSharp (reader 2). Fig 1 shows the screen shown by readers using the eye-tracking software.

### Study Radiography

The materials used in this study consisted of 440 joints (a set of 20 radiographs, including both hands and feet) of 10 patients with rheumatoid arthritis (ACR/EULAR 2010) chosen by a rheumatologist expert (AS) to represent the range of disease severity with early inflammatory rheumatism from the local cohort (from normal to severe damage). Using the mvdH, the score of erosions per hand was between 0 and 43, the number of erosions per foot was between 0 and 23, the score of Joint Space Narrowing (JSN) per hand was between 0 and 27, and the number of JSN per foot was between 0 and 19.

### Radiographic reading using the Sharp score

**Fig 2:**
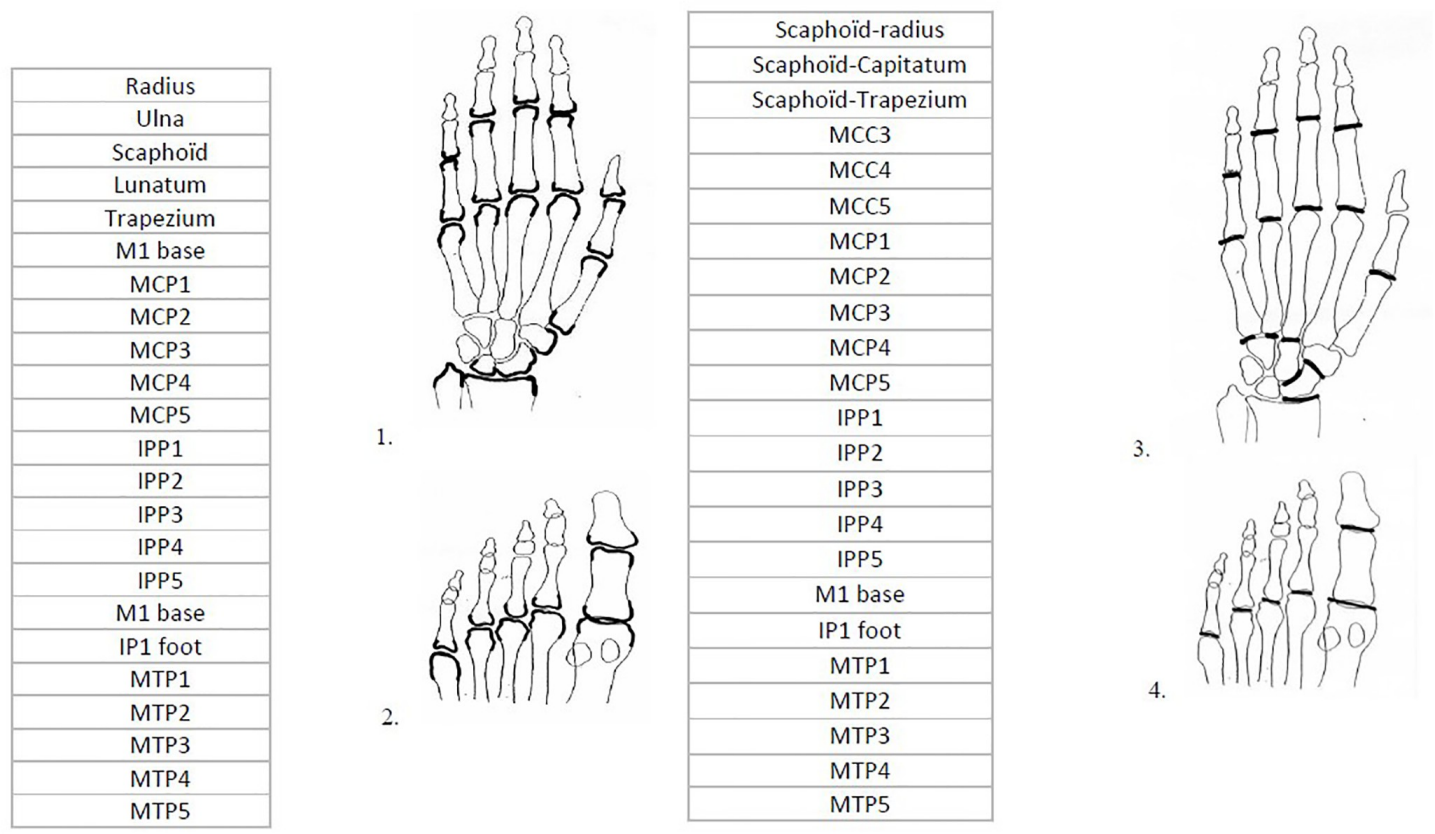
Joints involved in the modified van de Heijde Sharp score scores of erosions and JSN.

One reading was performed by Reader 2 according to the mvdH Sharp score for both erosions and JSN. This scoring lecture was considered the gold standard for our study to establish a reliable and accurate reference point for comparison. In total, each image was linked with a JSN Sharp score and erosion Sharp score. The joints involved in the scores of erosions and JSN are shown in Fig 2.

### Radiographic reading via eye tracking

The eye tracking reader (reader 1) trained to read radiographs with a metronome to learn to respect 2 seconds for one point of the mvdH Sharp score. Reader 1 was seated in front of a computer screen to interpret each radiograph using the eye-tracking score. Radiographs were read 4 times by an eye-tracking reader (2 times for the JSN eye-tracking score and 2 times for the erosion eye-tracking score).

For reader 1’s reading under metronome, we obtained an eye tracking score: a 1-point Sharp score was assigned every 2 seconds of fixations duration by the reader. Fixation duration is the time when the reader focuses on a anomaly without looking elsewhere. By dividing the fixation values by a pair of milliseconds by 1000 and then by two (with the reader fixed at 2 seconds for each erosion) and then subtracting the value from 0.5 (to eliminate the background noise of the duration of reading a normal joint), we obtained a reading in which 1 point was read by eye-tracking = 1 point for the mvdH Sharp score:

$$1\;pts\;mvdH=1\;pts\;Eyetracking\;mvdH=\frac{\frac{\left(\text{fixation}\;\text{values}\;\left(\text{ms}\right)\right)}{1000}}{2}-0.5$$

### Eye-tracking Software

An eye tracker consists of cameras, projectors and algorithms. The projectors create a pattern of near infra-red light on the eyes. The camera take images of the user’s eyes. Mathematical algorithms calculate the eyes’ positions and gaze point. Tobii Pro Lab Eye-tracking software, a validated, high-precision software package, was used to record gaze data at a sampling rate of 120 Hz and analyse the eye movements of the different readers. With this eye-tracking software, data values can be represented as colours in a map, diagram or image, also known as a heatmap. This software gives a good overview of what area of interest the reader is paying attention to by dividing the image into a grid within each pixel and showing the intensity of values in the recordings.

### Statistical analysis

440 joints were analysed (16 joints on each hand for erosions, 15 joints for JSN on the hands, and 6 joints on each foot). Eye tracking readings incorrectly captured by the eye tracker were considered no analysable. The data were analysed descriptively by calculating the fixation duration in milliseconds on each joint based on the diameter of the Gaze Plot’s circles created by the software. Gaze plots also show the location and order of the fixation duration at each point. The fixation time data are reported in a table that compares the recorded data with the initial Sharp score, which was established as the gold standard. The number of joints with at least one instance of erosion and JSN was analysed, and the local fixation time was converted to seconds. All the statistical analyses were performed using SPSS software version 25.0 (IBM, Armonk, NY) and were based on average values. We used the intraclass correlation coefficient (ICC) to determine intra-observer eye-tracking reliability (from 0.00 to 10.00). An ICC > 0.8 was considered excellent. For inter observer reliability, we performed an analysis by class (yes versus no and value from 0 to 10) using Cohen’s kappa.

## Results

### Radiographs evaluated

All 20 radiographs were read by both readers. A total of 440 joints were selected for interpretation. Finally, 416 and 396 joints could be interpreted when read by eye tracking for erosive involvement and JSN, respectively, because eye-tracking technology cannot measure the time spent when two pathological joints are too close together.

### mvdH Sharp score

On the 20 X-rays of rheumatoid arthritis patients analysed by eye tracking, the mean sharp score for erosions per joint was 0.52 +- 1.13, that for JSN per joint was 0.81 +- 1.36, and that for JSN and erosion was 2.27 +- 2.62 seconds and 1.78 +- 2.30 seconds, respectively. An analysis of the scores obtained by the eye-tracking method was performed jointly. The erosion sharp score was more important for the feet, notably for metatarsophalangeal (MTP) 3, MTP4 and MTP5, for which the mean length of reading was between 0.80 and 3.15 seconds. JSN was described in a diffuse way and was particularly high for metacarpophalangeal (MCP) 2, MCP3, MTP3, and MTP4, with a mean time of reading between 1.15 and 4.06 seconds. All corresponding results are presented in Table 1.

**Table 1:** Description of the global location and joint location (left and right) with sharp scores of erosion and JSN according to eye-tracking and the mean time of the readings. JSN: joint space narrowing, *MCP*: *metacarpophalangeal*, *MTP*: *metatarsophalangeal*, *MCC*: *metacarpocarpal; IPP interphalangeal*.

	Erosions			JSN		
	Location (right and left combined) of erosions	Score Sharp erosion (mean +- SD)	Means time of reading erosion in second (mean +- SD)	Location (right and left combined) of narrowings	Score Sharp narrowing (mean +- SD)	Means time of reading narrowing in second (mean +- SD)
**Global**	Global erosion	0.52 +- 1.13	1.78 +- 2.30	Global narrowing	0.81 +- 1.36	2.27 +- 2.62
**Hands**	Radius	0.25 +- 0.64	1.17 +- 1.04	Scaphoïd-radius	0.95 +- 1.40	4.06 +- 3.89
	Ulna	0.45 +- 0.89	1.93 +- 1.96	Scaphoïd-Capitatum	0.90 +- 1.02	2.35 +- 1.81
	Scaphoïd	0.40 +- 1.05	1.69 +- 2.65	Scaphoïd-Trapezium	0.75 +- 1.41	2.46 +- 2.65
	Lunatum	0.35 +- 0.75	1.53 +- 1.40	MCC3	0.75 +- 1.55	2.43 +- 2.78
	Trapezium	0.30 +- 0.57	1.68 +- 1.60	MCC4	0.35 +- 1.09	2.45 +- 3.98
	M1 base	0.35 +- 0.75	1.32 +- 1.35	MCC5	0.45 +- 0.95	1.84 +- 1.87
	MCP1	0.25 +- 0.55	1.11 +- 0.99	MCP1	0.40 +- 0.82	1.69 +- 1.79
	MCP2	0.90 +- 1.12	2.39 +- 2.47	MCP2	1.50 +- 1.54	3.06 +- 2.63
	MCP3	0.80 +- 1.12	2.62 +- 3.42	MCP3	1.40 +- 1.47	3.06 +- 2.65
	MCP4	0.30 +- 0.66	1.26 +- 1.51	MCP4	0.85 +- 1.39	1.88 +- 2.40
	MCP5	0.10 +- 0.31	0.80 +-0.71	MCP5	0.85 +- 1.53	2.18 +- 2.94
	IPP1	0.15 +- 0.67	1.02 +- 1.38	IPP1	0.30 +- 0.80	1.15 +- 1.36
	IPP2	0.45 +- 0.89	1.44 +- 1.73	IPP2	0.60 +- 1.19	1.50 +- 1.90
	IPP3	0.50 +- 0.83	1.50 +- 1.53	IPP3	0.65 +- 1.22	1.80 +- 2.25
	IPP4	0.30 +- 0.58	1.26 +- 1.13	IPP4	0.65 +- 1.35	1.77 +- 2.62
	IPP5	0.30 +- 0.89	1.84 +- 1.91	IPP5	0.55 +- 1.10	1.61 +- 2.12
**Feet**	M1 base	0.35 +-0.75	1.32 +- 1.34	M1 base	0.45 +- 0.95	1.84 +- 1.87
	IP1 foot	0.55 +-0.93	2.03 +- 1.78	IP1 foot	0.90 +- 1.25	2.39 +- 2.50
	MTP1	0.75 +- 2.05	1.94 +- 2.61	MTP1	0.70 +- 1.49	2.00 +- 2.48
	MTP2	0.25 +- 0.64	1.20 +- 1.19	MTP2	0.80 +- 1.54	2.07 +- 2.81
	MTP3	1.20 +- 1.99	3.10 +- 3.67	MTP3	1.25 +- 1.80	3.65 +- 3.45
	MTP4	1.10 +- 2.08	2.93 +- 4.20	MTP4	1.45 +- 1.73	3.32 +- 3.37
	MTP5	1.15 +- 1.73	3.15 +- 4.02	MTP5	0.80 +- 1.32	1.91 +- 2.26

### Agreement between eye tracking mvdH Sharp score and classical mvdH Sharp score

Table 2 shows the ***a***greement between eye tracking mvdH Sharp score and classical mvdH Sharp score for both erosion (upper) and JSN (lower). The intraclass agreement of the eye tracking method was excellent at 0.90 (95% CI 0.88–0.92) for both erosions and JSN. The agreement between eye tracking mvdH Sharp score and classical mvdH Sharp score by class yes (at least one erosion or JSN) versus no (no erosion or no JSN) was excellent for both erosions (kappa 0.82; 95% CI: 0.79–0.0.85) and JSN (kappa: 0.68; 95% CI: 0.65–0.0.71). The agreement between eye tracking mvdH Sharp score and classical mvdH Sharp score by class (0 to 10) remained excellent for erosions (kappa 0.82; 95% CI: 0.79–0.0.85) and good for JSN (kappa: 0.68; 95% CI: 0.65–0.71).

**Table 2:** Agreement between eye tracking mvdH Sharp score and classical mvdH Sharp score for both erosion (upper) and JSN (lower). *JSN*: *joint space narrowing*.

	***Classical mvdH Sharp score* Erosion**							
		0	1	2	3	4	5	≥6
***Eye tracking mvdH Sharp score* Erosion**	0	307	1	0	0	0	0	0
	1	3	35	3	0	0	0	0
	2	0	13	33	1	0	0	0
	3	0	0	0	6	1	0	0
	4	0	0	0	0	1	4	0
	5	0	0	0	0	0	1	3
	≥6	0	0	0	0	0	1	1
	**Total**	307	1	0	0	0	0	0
	***Classical mvdH Sharp score*** JSN							
		0	1	2	3	4	5	≥6
***Eye tracking mvdH Sharp score* JSN**	0	261	2	1	1	0	0	0
	1	4	19	1	0	0	0	0
	2	0	14	14	0	1	0	0
	3	1	3	20	20	0	0	1
	4	0	0	5	11	16	0	1
	5	0	0	0	0	0	0	0
	≥6	0	0	0	0	0	0	0
	**Total**	307	1	0	0	0	0	0

### Heatmap results

**Fig 3:**
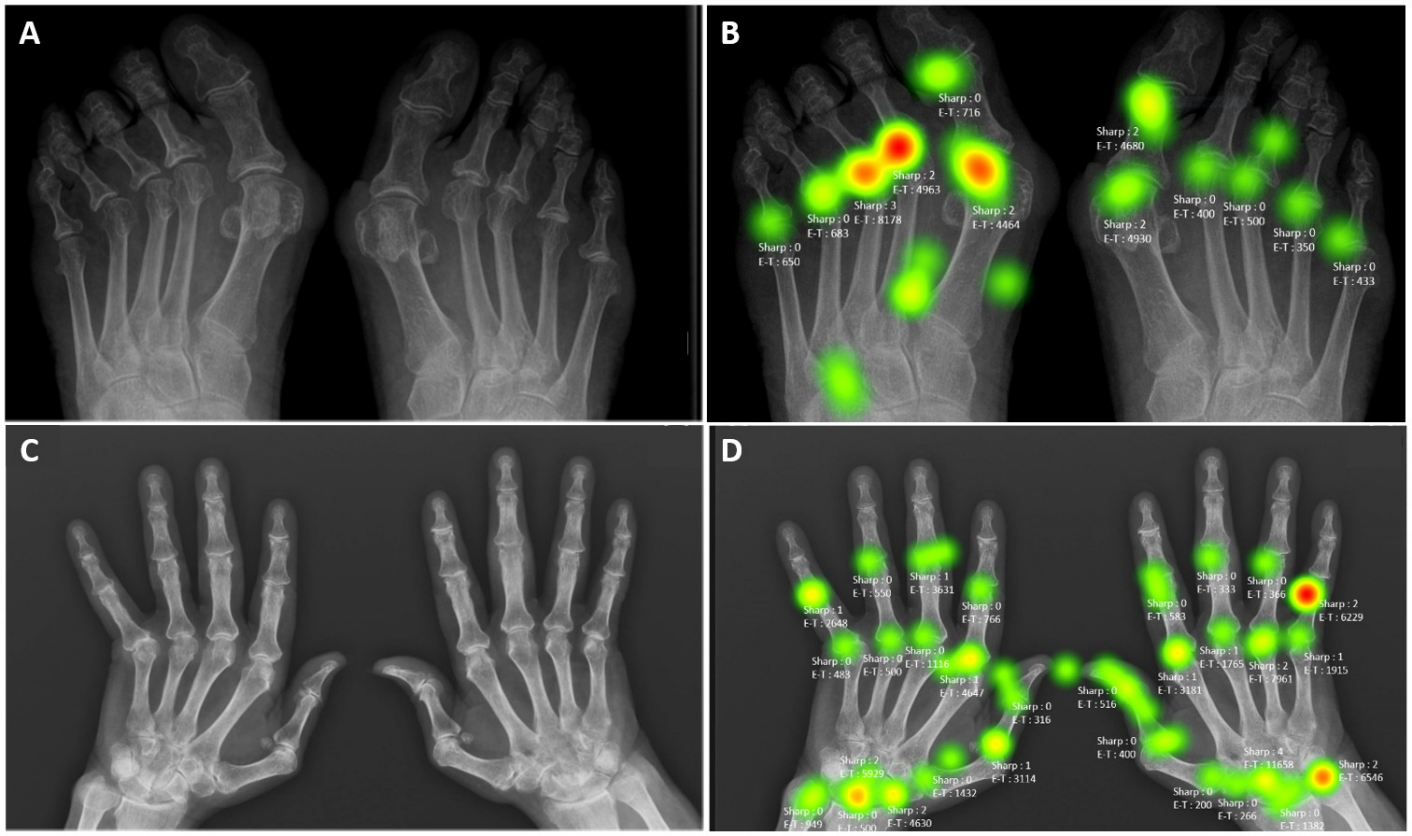
Example of a foot and hand erosion patient with a modified van der Heijde Sharp score (value written for each joint after Sharp) and eye-tracking score (value written for each joint after E-T and color green to red according to the duration) in milliseconds.

Heatmaps were generated to reveal which subregions tended to contribute to severe lesions. Both heatmaps and glaze plot figures of each reading show clear segmentation and fixation duration. Joints with a “hot” colour had the highest Sharp score and fixation duration, and conversely, “cold” (green) colours represented a short fixation time and a low severity of joint damage. According to the statistical analysis, the colour of the joint was strongly correlated with the severity of the sharp spot, which was preliminarily established via the gold standard. Green pixels for Sharp lower than 1, yellow for Sharp equal to 1, and orange to red for Sharp above or equal to 2. Fig 3 shows an example of a foot and hand erosion of one patient with a modified van der Heijde Sharp score and eye-tracking score (E-T) in milliseconds.

## Discussion

### Main findings

The aim of this study was to develop a technique using eye-tracking software to analyse radiographs of rheumatoid arthritis patients, segment the joints and establish a score of lesions with the fixation time score and pixels at the same time. We found that his new method of quantifying the mvdH score based on readings via eye-tracking technology is highly correlated with the classical mvdH Sharp score determined by an experienced radiologist. This method allowed us to segment and evaluate the joint lesions one time because of the fixation duration.

### Interest of eye-tracking technology for X-rays reading

Our findings suggest that eye tracking can provide valuable insights into the processes of radiographic interpretation. These findings highlight the importance of sharp score training to best evaluate joints, particularly for the accurate diagnosis and treatment of rheumatologic disorders. This approach allowed us to identify erosions and narrow more comprehensively with a score translation with colours on heatmaps. It would be a more intelligible and playful technique to illustrate the severity of lesions to students, professionals or patients. This method is also less time-consuming; instead of a two-phase lecture with score analysis and segmentation, this technique makes it possible to merge the 2 phases into one, save time and reduce the number of professionals involved.

### Use of IA for analysing radiographic images

In the literature, deep learning and convolutional neural networks are the most commonly used artificial intelligence methods for analysing radiographic images. Few studies using IAs have been described in the literature; these studies used the Sharp/van der Heijde SvdH score, erosions score, JSN, or both, with variable sensitivity and specificity [24,25,26]. Recently, Wang et al. described an artificial intelligence study of X-ray images scored by the Sharp/van der Heijde score index trained with You Only Look Once version 4 (YOLOv4), an object detection model trained to look at an image and search for a subset of object classes [24]. By understanding the similarities between the variations in the sharp score and the heatmap created by the fixation duration, AI could directly create a heatmap score for an easier and more comprehensive interpretation of radiographic findings. Our study also revealed interesting insights regarding the impact of reading time on radiographic interpretation, shedding light on its relationship with diagnostic accuracy. The experimental data were subsequently analysed with artificial intelligence software before training to read the rheumatoid arthritis radiographs of several cohorts. The correlation between Sharp and the colour score of heatmaps was determined to analyse future radio files with a colour code more explicit than the van der Heijde modified Sharp score. This approach will be very intuitive for students or professionals who are less accustomed to the radiology discipline. One limitation of our study is the definition of the gold standard, which was based on the means of reading by one experienced reader.

### Limits

This approach could introduce bias, as the gold standard may not necessarily reflect the true or objective interpretation of the radiographs; rather, the differences in reading, intraobservers and interobserver agreements between eye-tracking and sharpeners are similar to the differences that could occur between 2 radiologists interpreting images.

Additionally, the sample size was relatively small, which could limit the generalizability of the findings to larger populations. Our study focused only on a simplified radiographic score, which may not fully capture the complexity of radiographic interpretation in clinical practice. Furthermore, the steps of reasoning by an AI must be clearly described to clinicians, but often, these algorithms include "black boxes", complex information processing steps that make very little sense to a human. However, eye tracking technology is still relatively new, and limitations exist regarding its use. For example, eye tracking can be affected by factors such as lighting, screen resolution, participant comfort, and glasses worn, which may affect the quality of the data collected. With respect to the efficiency of the software, there may be limitations in wrist fixation problems. Since the joint bonds are very close together, the fixing zones are regularly confused. Therefore, while eye tracking is a promising tool for studying radiographic interpretation, it should be used in conjunction with other methods to provide a more comprehensive understanding of the cognitive processes involved.

Another limitation of our study is that we conducted it in only one center, with one eye tracking reader and one experimented radiologist as gold standard. Nevertheless, the radiologist (FG) is highly experimented, with a very high agreement with other specialist of mvdH Sharp score, and the eye tracking reader was trained with previous reading.

### Conclusion

In conclusion, our study demonstrated the usefulness of eye-tracking technology for evaluating severity through radiographic interpretation strategies, segmenting joints and establishing a score for lesions based on both the fixation time score and the number of pixels. It would be a more intelligible and playful technique to illustrate the severity of lesions to students, professionals or patients. Our findings may have important implications for improving interdisciplinary communication and collaboration in the diagnosis and treatment of rheumatological disorders. In the future, this technique could also be developed for other disciplines using scores in their diagnostics.

## Supporting information

S1 Data File reporting readings. (XLS)
